# Solitary metastasis to the breast after complete resection of encapsulated type AB thymoma: a case report

**DOI:** 10.1186/s13256-015-0551-6

**Published:** 2015-03-24

**Authors:** Pei-Wen Huang, Kuo-Ming Chang

**Affiliations:** Department of Pathology, Mackay Memorial Hospital, Hsinchu, No.690, Sec. 2, Guangfu Rd, East Dist Hsinchu City, 30071 Taiwan

**Keywords:** Breast metastasis, Encapsulated thymoma, Type AB

## Abstract

**Introduction:**

Type AB thymoma is generally considered a benign tumor with excellent prognosis. Rare case reports of type A and AB thymomas with recurrence and metastasis have been documented, although extrathoracic metastases are extremely rare. To the best of our knowledge, this is the first reported case of type AB thymoma with solitary metastasis to the breast.

**Case presentation:**

We describe an 83-year-old Taiwanese woman with a metastatic thymoma to the breast 10 years after complete resection of noninvasive and encapsulated primary tumor, and analyze the possible factors to explain the recurrence and metastasis of the stage I thymomas.

**Conclusions:**

Even a clinically benign tumor such as type AB thymoma still has a possibility of metastasizing to an unusual site. When any uncommon tumor presents in any site, a suspicion of secondary neoplasm and thorough clinical history are required.

## Introduction

Thymomas are neoplasms that arise from or exhibit differentiation towards thymic epithelium. The World Health Organization (WHO) classification of thymomas is based on the morphology of epithelial cells as well as the lymphocyte-to-epithelial cell ratio, and thymomas are divided into five groups (type A, AB, B1, B2, and B3). In general, type A, AB and B1 thymomas are considered no/low-risk groups of tumors with better prognosis than type B2 and B3 thymomas which are moderate/high-risk tumors [1]. However, rare case reports of type A and AB thymomas with recurrence and metastasis have been documented, and extrathoracic metastases are extremely rare. We describe a case with stage I and type AB thymoma, which metastasized to the breast 10 years after complete resection of primary tumor.

## Case presentation

### Clinical summary

**Figure 1:**
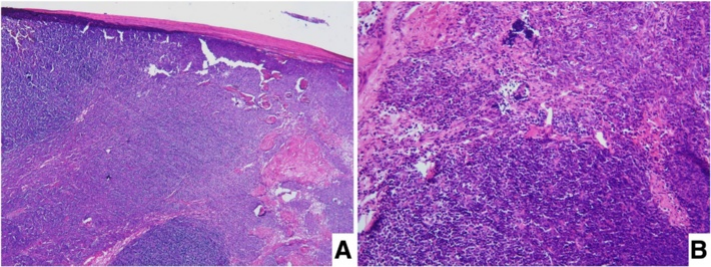
**Primary thymoma. (A)** The primary thymoma is well-encapsulated without pericapsular invasion. **(B)** The primary tumor is composed of a lymphocyte-poor type A thymoma component and lymphocyte-rich type B component.

**Figure 2:**
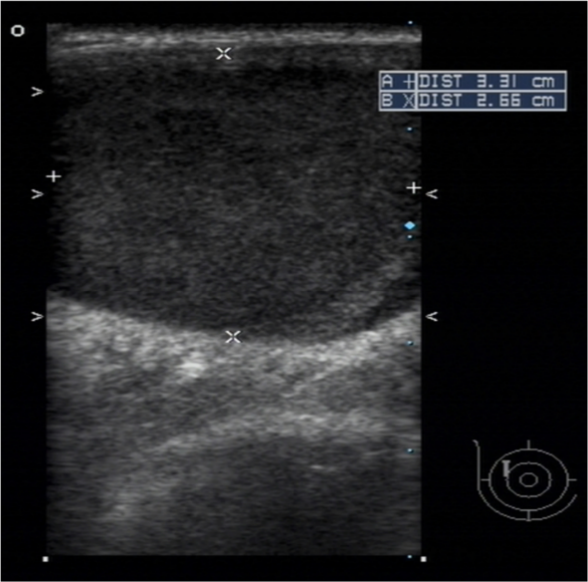
**Mammography reveals a well-defined hypoechoic oval mass in right breast, occupying the whole thickness of breast, and measuring 4.0×3.2×2.6cm in size.**

An 83-year-old Taiwanese woman found a palpable mass in her right breast for 1 year. The lesion was approximately 1cm in greatest dimension last year and had got larger in the past 6 months. She had a history of type AB thymoma and was treated with complete tumor resection 10 years ago. The thymoma was 15cm in greatest dimension, encapsulated with focal pericardial adhesion and without pericapsular invasion (Figure 1). A mammography revealed a well-defined hypoechoic oval mass in 10 o'clock direction and 3.0cm away from her nipple, occupying the whole thickness of her breast, and measuring 4.0×3.2×2.6cm in size (Figure 2). A tumor excision was performed.

### Pathological findings

**Figure 3:**
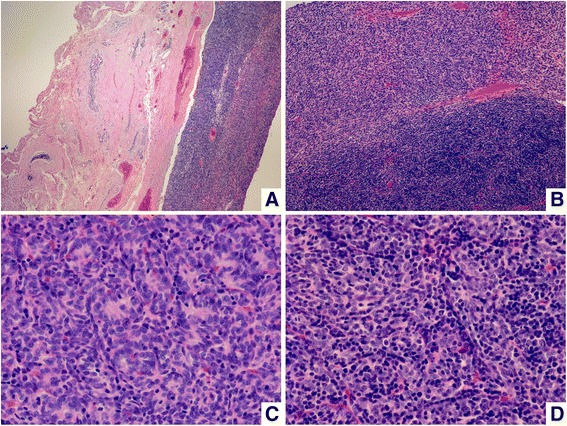
**Breast tumor. (A)** The tumor is well encapsulated and atrophic ductal components are on the left. **(B)** It is composed of two different tumor components and admixed with variable amounts of lymphocytes. **(C)** Some neoplastic cells arranged in rosettes without lumen in the lymphocyte-poor background. **(D)** The most neoplastic cells are oval to polygonal or short fusiform with small round-to-oval, pale nuclei and no or inconspicuous nucleoli, set in the lymphocyte-rich background.

**Figure 4:**
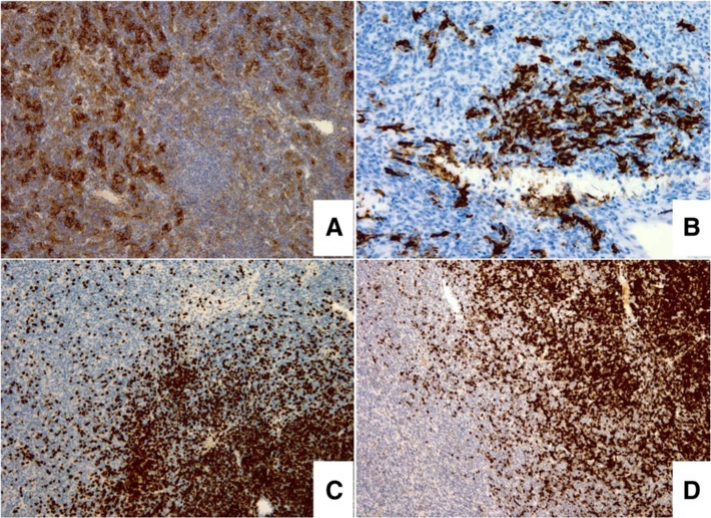
**Immunohistochemistry. (A)** The neoplastic cells are diffusely positive for cytokeratin, and **(B)** few are also positive for CD20. The lymphocytes are positive for **(C)** CD3 and **(D)** CD1a.

The specimen consisted of a piece of breast tissue partially occupied by an oval encapsulated mass that measured 5.0×3.8×3.5cm in size, with focal artificial disruption. The cut surface of the tumor was tan red and the focal was light yellowish. The tumor consistency was soft and fragile. On microscopic examination, the well-encapsulated tumor was composed of neoplastic cells arranged in solid sheets, vague fascicles or ribbons and few rosettes without lumen, and admixed with variable amounts of lymphocytes (Figure 3A-C). The neoplastic cells set in the lymphocyte-rich background were oval, polygonal or short fusiform, and had small round-to-oval nuclei with inconspicuous nucleoli (Figure 3D). The spindle tumor cells were mainly seen in the lymphocyte-poor area. The two groups of neoplastic cells either separated with a discrete border or were intermixed together. Mild nuclear atypia was occasionally seen. Mitoses were scanty. Some aggregates of histiocytes and few dilating and mildly branching thin-walled vessels were also found. The neoplastic cells were diffusely positive for cytokeratin (Figure 4A), partially positive for vimentin and smooth muscle actin, mostly positive for p63, and focally immunoreactive with epithelial membrane antigen. A few tumor cells were also positive for CD20 (Figure 4B). Some spindle neoplastic cells were surrounded by collagen IV deposition. The tumor cells were negative for CD21 and CD34. The lymphocytes were positive for CD3, CD5, CD1a, and CD99 (Figure 4C-D). Few scattered individual and small groups of interdigitating reticulum cells were seen by S-100 protein staining. From these features and past history, we diagnosed this tumor as a metastatic type AB thymoma to the breast.

## Discussion

We reported a case with metastatic type AB thymoma to the breast 10 years following the complete resection of primary noninvasive thymoma. The postoperative recurrence rate for noninvasive thymoma has been reported to be 0 to 7%, and the majority is local recurrence [2]. WHO type A and AB thymomas are generally regarded as clinically benign neoplasms. However, some studies document recurrence, metastases and mortality in patients with these two subtypes [3]. The metastatic sites of type A and AB thymomas include pleura, lung, liver, brain, peritoneum, kidney and bone. A mean time interval of 62.25 months (range: 12 to 138 months) between diagnosis of primary tumor and recurrence or metastasis has been reported [3].

Metastases from extramammary malignancy to the breast are also rare and account for approximately 0.3 to 2.7% of all malignant mammary tumors, and less than 500 cases have been reported [4]. Metastatic thymic tumors of the breast are extremely rare. To the best of our knowledge, only two cases with well-documented metastatic thymomas to the breast have been reported in the English literature. One of these had inoperable primary type B3 thymoma and was treated only with chemotherapy, and metastatic tumor to the breast occurred in 2 years [5]. The other case had a complete resection for noninvasive thymoma without metastasis 20 years ago. With a 19-year interval, a pulmonary metastasis of type B3 thymoma with pleural invasion developed, then the breast metastatic tumor occurred 1 year later [6]. In our case, the patient had neither advanced disease at the presentation nor pulmonary involvement before breast tumor occurring. She had a solitary metastatic tumor in her right breast and no other recurrence or metastatic sign shown in the postoperative image and clinical studies.

Some factors have been proposed to explain the recurrence and metastasis of stage I thymomas. Previous series studies revealed intraoperative adhesion of noninvasive thymomas had a significantly higher recurrent rate [7,8]. The proliferation of fibrovascular tissue during fibrosis might increase the risk of lymphovascular permeation and the possibility of recurrence and metastasis. A tumor size of 8cm or larger in thymic epithelial tumors was supposed to be an adverse prognostic factor [9]. The large tumor size might increase the probability of missing some areas with higher-grade histological type or microinvasion due to inadequate sampling. Transformation of low-grade to high-grade histology has also been reported as a cause of recurrence and metastasis [10]. In our case, pericardium adhesion and large tumor size (>8cm) were noted in the primary tumor, but tumor capsule or pericardium invasion was absent, and the histological features of metastatic and primary tumors were identical. Some studies have suggested that molecular analysis may be useful for prediction of tumor behavior, and it needs further research.

## Conclusions

Although extrathoracic metastatic thymoma and metastases from extramammary malignancy to the breast are rare, accurate diagnosis is important to avoid unnecessary treatment. Besides pathologic and immunohistochemical findings, the clinical history is crucial for differential diagnosis. Even a clinical benign tumor such as type AB thymoma still has a possibility of metastasizing to an unusual site. When any uncommon tumor presents in any site, a suspicion of secondary neoplasm and thorough clinical history are required.

## Consent

Written informed consent was obtained from the patient for publication of this Case Report and any accompanying images. A copy of the written consent is available for review by the Editor-in-Chief of this journal.

## Abbreviation

WHO: World Health Organization
